# To Stent or Not to Stent: Is It a Question? Routine Trans‐Cystic Stenting Does Not Reduce Biliary Anastomotic Strictures Post‐Liver Transplantation

**DOI:** 10.1111/ans.70224

**Published:** 2025-06-23

**Authors:** Marcos Vinicius Perini, Eunice Lee, Michael Fink, Graham Starkey, Osamu Yoshino, Bartholomew McKay, Ruelan Furtado, Enes Makalic, Robert Jones

**Affiliations:** ^1^ Department of Surgery (Austin Precinct) The University of Melbourne Heidelberg Australia; ^2^ HPB & Liver Transplant Surgery Unit Austin Health Heidelberg Australia; ^3^ Victorian Liver & Intestinal Transplant Unit Austin Health Heidelberg Australia; ^4^ The University of Melbourne, Melbourne School of Population and Global Health Carlton Australia

**Keywords:** biliary anastomotic stricture, biliary complications, biliary stent, liver transplantation, trans‐cystic stent

## Abstract

**Background:**

We aim to compare the incidence and risk factors for biliary anastomotic stricture (BAS) in patients undergoing orthotopic liver transplant (OLT) with and without transcystic externalised trans‐anastomotic biliary stenting.

**Methods:**

A retrospective analysis was performed of a prospective database focused on 836 cadaveric OLT. Primary outcome measures were the incidence of BAS and risk factors related to its development.

**Results:**

Duct‐to‐duct anastomosis was the most commonly performed biliary reconstruction (90.5%). Transcystic externalised trans‐anastomotic biliary stenting was performed in 420 patients (62.0%), being mostly used in patients having a duct‐to‐duct anastomosis (63.6%). BAS was seen in 222 (32.8%) patients, with a median time to diagnosis of 145.5 days (IQR 50.3–370.5). BAS was higher in patients with a duct‐to‐duct reconstruction when compared to those having a bilio‐enteric reconstruction (34.3% vs. 18.7%, *p* = 0.02). The prevalence of BAS was not significantly different between patients who were stented and those who were not (34.5% vs. 30.0% respectively, *p* = 0.25). Multivariable analysis showed that older donor age, transplants performed earlier in the study period, higher MELD score, and type of biliary reconstruction (duct‐to‐duct) were independently associated with a higher risk of BAS.

**Conclusion:**

Transcystic externalised biliary anastomotic stenting is not associated with a reduced biliary stricture incidence in OLT.

## Graphical abstract

Biliary anastomotic stricture (BAS) occurs with variable incidence following orthotopic liver transplantation. This study aimed to assess the risk factors for BAS in a large cohort and showed that routine trans‐cystic biliary stenting had no impact on its development.

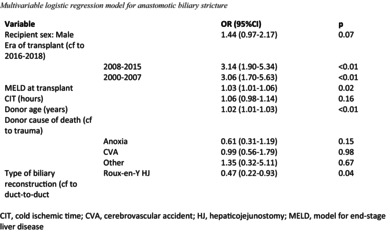

## 1 Introduction

Orthotopic liver transplant (OLT) is the standard treatment for terminal liver disease and hepatocellular cancer associated with cirrhosis in selected cases [1]. Since the first liver transplant performed by Starzl, surgeons have faced the complications of biliary reconstructions with variable incidence. Even when performing a semi‐elective choledocho‐choledochostomy with viable tissue and an adequate surgical technique, the development of a benign biliary anastomotic stricture (BAS) is still a reality [2, 3]. In order to decrease its incidence, biliary drainage has been proposed more in accordance with surgeon speculation than published data [4]. Few prospective trials have been conducted to date with conflicting data, and surgeons continue to debate this issue, illustrating its controversy [5, 6, 7, 8, 9, 10].

Theoretical advantages of biliary stenting are prompt access to the biliary tree for radiological studies, decreased intra‐biliary pressure, bile output assessment, and reduced BAS rate [11, 12]. On the other hand, biliary stenting means that at some point surgeons should remove it, exposing the patient to complications such as bile leakage, peritonitis, and/or reoperation, particularly if the stent is externalised. Surgeons that argue against stent placement suggest these complications are a major cause of morbidity, increase hospital costs, and significantly impact patient recovery [5, 6, 7, 8, 9].

Some reports have shown feasibility and low morbidity with the use of transcystic trans‐anastomotic biliary stenting [13] but there exist few series of patients reported in the literature [14, 15]. By placing a trans‐anastomotic stent through the cystic duct (rather than choledochotomy), a natural orifice is used to exteriorize the stent. Moreover, the gallbladder peritoneal attachment can be used to wrap around the stent, creating natural protection if biliary leakage happens when the stent is removed [13].

Based on these unanswered questions and the controversy surrounding biliary stenting, we aim to compare the incidence and risk factors for BAS in patients submitted to OLT with and without transcystic externalised trans‐anastomotic biliary stenting.

## 2 Patients and Methods

### 2.1 Study Design

This retrospective analysis of a prospective database was conducted on patients undergoing OLT between 1st January 2000 and 31st December 2018 at the Victorian Liver and Intestinal Transplant Unit, Austin Health, Victoria, Australia. Donor and recipient data on demographics, laboratory data, operative parameters, postoperative course, and recipient overall survival and graft survival were collected. Data was reviewed for patients with BAS and compared with patients who did not develop BAS. The primary outcome was the development of BAS at any time during follow up. BAS was defined as any stricture or irregularity of the extra‐hepatic duct involving the biliary anastomosis, in the presence of a patent hepatic artery, requiring treatment (dilatation, stenting, or both). Strictures were diagnosed on radiology or during endoscopic procedures and were required to have an associated cholestatic derangement on liver function testing. Radiological modalities used for diagnosis were Magnetic Resonance Cholangiopancreatography (MRCP), Endoscopic Retrograde Cholangiopancreatography (ERCP), Percutaneous Trans‐hepatic Cholangiography (PTC) and biliary catheter cholangiogram. In all patients with BAS, hepatic artery thrombosis was excluded using either Doppler ultrasound or contrast enhanced CT scan. For the purposes of this study, recipient diseases were classified into acute hepatic necrosis (AHN), hepatitis (hepatitis B and C), malignancy (hepatocellular carcinoma), PSC (primary sclerosing cholangitis), PBC (primary biliary cirrhosis) and other (alcohol, cryptogenic, metabolic, NASH). Ethical approval for the study was provided by the institutional ethics committee (HREC/91948/Austin‐2022).

### 2.2 Exclusion Criteria

All transplants from donation after circulatory death (DCD) were excluded from the analysis due to the higher incidence of biliary complications. Patients who died within 90 days, split grafts, pediatric transplants, and transplants in which data on the type of anastomosis were not recorded were also excluded. In cases of re‐transplantation within 90 days, the first transplant was excluded from the analysis. In cases of late re‐transplants, both transplants were recorded, and the follow up period was censored at the time of re‐transplant. Patients presenting with BAS and non‐anastomotic biliary strictures were also excluded due to uncertainty of the cause.

### 2.3 Surgical Procedure

All donor organs were procured from deceased donors who were ABO compatible with the recipient. Organ procurement was performed using standardized techniques and, in all cases, a pre‐flush of low viscosity solution via the aorta was performed. Ex situ perfusion at the donor back table was done using the final perfusion solution (University of Wisconsin or Histidine‐Tryptophan‐Ketoglutarate). All perfusion was performed under gravity feed. Implantation was via a standard piggy‐back approach, as described elsewhere [16]. Duct‐to‐duct biliary anastomosis was the preferred reconstruction technique, and the technique used was at the surgeon discretion (running, interrupted or a combination). Patients with PSC underwent a bilio‐enteric anastomosis because of the presence of large‐duct strictures or a duct‐to‐duct anastomosis where the recipient's extra‐hepatic bile duct was normal. Routine trans‐anastomotic biliary stents were placed via the cystic duct, when feasible. Usually, these were size 6 Fr infant feeding catheters placed trans‐cystically across the biliary anastomosis to allow access to the biliary tree post‐transplant, but size did depend on the biliary tract diameter and anatomy (in cases of lower cystic duct insertion, it is not possible to place the stent). The stent was secured at the cystic duct with dissolvable sutures, and the gallbladder peritoneal surface was used to wrap around the stent, which was exteriorized in the right upper quadrant on free drainage. Patients having a bilio‐enteric anastomosis had the trans‐anastomotic stents placed and exteriorized distally in the Roux en *Y* limb around 30 cm from the anastomotic site. The transplant surgical team remained constant for the duration of this study, and there were no major changes in operative technique throughout the study period.

### 2.4 Post‐Operative Management

The standard immunosuppression regimen within our unit is described elsewhere [17]. Biliary catheters were clamped after biliary imaging was obtained usually between POD 3 to POD 5. At 3 months post operatively, a check cholangiogram was performed to study the biliary tree and assess contrast flow to the duodenum. The stent was then removed in the outpatient clinic if no significant abnormalities were found. Patients without biliary catheters would only be investigated with radiology if clinical suspicion of a stricture was raised.

### 2.5 Data Collection and Analysis

The variables considered were chosen according to a literature search of factors associated with the development of BAS. Groups were compared using the Wilcoxon rank‐sum test for continuous variables and chi‐square test for categorical variables. Survival analysis was performed using Kaplan Meier estimates, and survival between the two groups was compared using the log‐rank test. A *p*‐value of < 0.05 was considered significant.

Risk factors for the development of BAS were identified using univariate logistic regression. Variables with a *p*‐value of less than 0.1 were selected for inclusion in a multivariable logistic regression model. The discrimination performance of the model was assessed by the area under the receiver operating curve (AUROC), with internal cross‐validation of the AUROC performed 100 times using random 80:20 training to test data splits. Analysis looking at the secondary outcome of early BAS (diagnosis prior to 1 year follow up post‐transplant) was performed on all patients followed up for at least 1 year, using the same methods as detailed above.

## 3 Results

### 3.1 Prevalence and Characteristics of Patients With Biliary Anastomotic Stricture

From the 836 transplants performed, 677 patients were included. The median age at transplant was 53.9 years (IQR 46.0–60.0) and males accounted for 70.0% of transplants.

The median follow‐up time was 5.5 years, ranging from 80 days to 18.9 years.

A total of 222 patients (32.8%) undergoing OLT developed BAS, with a median time to diagnosis of 145.5 days (IQR 50.3–370.5). Of the patients who were diagnosed with BAS, 166 (74.8%) developed BAS after 1 year, and 74 (33.3%) developed BAS within 90 days of OLT. Patients with a diagnosis of primary sclerosing cholangitis (PSC) had a trend toward a slightly lower rate of BAS (25.0% vs. 32.7%, *p* = 0.21). However, almost half of the recipients with PSC had bilio‐enteric reconstruction (46.9%, *n* = 30).

The BAS rate was higher for patients who underwent a duct‐to‐duct reconstruction compared to Roux‐en‐Y (34.3% vs. 18.7%, *p* = 0.02). Although there was a higher BAS rate for patients who had anastomoses performed with a continuous suture technique, this difference did not reach significance (33.4% vs. 26.6%, *p* = 0.13).

Biliary stenting was performed in 420 patients (62.0%), the majority of whom had a duct‐to‐duct anastomosis (93%, *n* = 390). The prevalence of BAS was not significantly different between patients who were stented and those who were not (34.5% vs. 30.0% respectively, *p* = 0.25) (Table 1).

**TABLE 1:** Donor, surgical, and recipient characteristics of patients with and without anastomotic biliary stricture.

		Without AS *n* = 455	With AS *n* = 222	Total *n* = 677	*p*
**Donor factors**					
Age (years)		45.0 (30.0–57.0)	51.0 (38.2–60.0)		< 0.01
Sex					0.12
	Female	197	111	308	
	Male	258	111	369	
Cause of death					0.01
	CVA	257	157	414	
	Anoxia	100	27	127	
	Trauma	64	29	93	
	Other	7	4	11	
	NA	27	5	32	
CIT (hours)		6.3 (5.2–7.9)	6.7 (5.3–8.5)		0.03
	NA	3	1	4	
CMV risk					0.01
	Low	362	177	539	
	High	83	29	112	
	NA	10	16	26	
**Surgical factors**					
Biliary stent					0.25
	No	180	77	257	
	Yes	275	145	420	
Type of biliary reconstruction					0.02
	Duct to duct	403	210	613	
	Roux en Y	52	12	64	
Suture technique					0.09
	Continuous	233	117	350	
	Interrupted	114	44	158	
	Continuous and interrupted	21	5	26	
	NA	87	56	143	
**Recipient factors**					
Disease					0.72
	AHN	29	10	39	
	Hepatitis B/C	103	58	161	
	Malignancy	84	40	124	
	PBC	16	10	26	
	PSC	48	16	64	
	Other	175	88	263	
Age (median/IQR)		54.1 (26.0–60.0)	53.0 (22.0–58.1)		0.14
Sex					0.04
	Female	150	55	205	
	Male	305	167	472	
MELD at transplant		10 (8–12)	10 (8–13)		< 0.01
Era of transplant					< 0.01
	2016–2018	108	19	127	
	2008–2015	221	115	336	

Abbreviations: AHN, acute hepatic necrosis; BAS, biliary anastomotic stricture; CIT, cold ischemic time; CMV, cytomegalovirus; CVA, cerebrovascular accident; MELD, model for end‐stage liver disease; PBC, primary biliary cirrhosis; PSC, primary sclerosing cholangitis; WIT, warm ischemic time.

There were some significant differences in the characteristics of patients who developed BAS compared to those who did not, including recipient sex, year of transplant, type of biliary reconstruction, and MELD score at transplant. Median donor age and cold ischemic time (CIT) were also significantly higher in patients who developed BAS.

### 3.2 Management of Biliary Anastomotic Stricture

Of the 222 patients who developed BAS, 91.4% (*n* = 203) were managed with ERCP; 6.3% (*n* = 14) with percutaneous trans‐hepatic biliary drainage (PTBD) and ERCP; and 2.3% (*n* = 5) with PTBD alone. A single treatment episode was required in 21.6% of patients (*n* = 48) usually involving a sphincterotomy and pneumatic balloon dilatation, with a further 54.1% of patients (*n* = 120) requiring between two and five interventions, usually due to progressive dilatation and/or stent manipulation. Approximately one quarter of patients (24.3%, *n* = 54) required more than five interventions.

### 3.3 Survival Analysis

**FIGURE 1:**
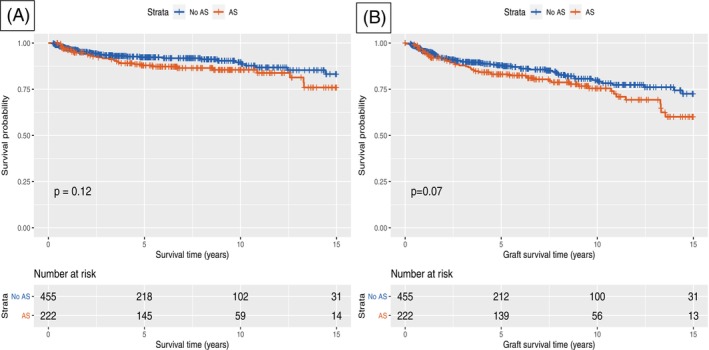
Kaplan Meier survival curves; (A) Overall survival and (B) Graft survival of patients with and without biliary anastomotic stricture (AS).

There were no significant differences in unadjusted overall survival or graft survival between patients who developed BAS and those who did not (log rank *p* = 0.12 and 0.07, respectively) (Figure 1). The 10‐year overall and graft survival for patients with and without BAS was 85.5% versus 89.6%, and 75.5% versus 79.9%, respectively. Of note, a tendency in reduced graft survival can be seen in Figure 1B in patients having a biliary stricture; however, that was not statistically significant (*p* = 0.07).

### 3.4 Risk Factors for the Development of Biliary Anastomotic Stricture

Using univariate logistic regression, eight variables were found to be associated with BAS risk, shown in Table 2. The multivariable model showed that older donor age, transplants performed earlier in the series, higher MELD score, and surgical anastomotic type (duct‐to‐duct) remained independently associated with a higher risk of BAS (Table 3). Recipients with an identical blood group to the donor also had a higher risk of BAS, compared to compatible or incompatible blood groups.

**TABLE 2:** Univariate analysis of risk factors for anastomotic biliary stricture.

Variable		OR (95% CI)	*p*
Recipient sex: Male		1.49 (1.04–2.16)	< 0.01
Era of transplant (cf to 2016–2018)			< 0.01
	2008–2015	3.20 (2.00–5.20)	< 0.01
	2000–2007	3.44 (2.07–5.83)	< 0.01
MELD at transplant		1.05 (1.02–1.07)	0.05
CIT (hours)		1.07 (1.00–1.14)	< 0.01
Donor age (years)		1.02 (1.01–1.03)	< 0.01
Donor cause of death (cf to trauma)			< 0.01
	Anoxia	0.60 (0.32–1.10)	0.10
	CVA	1.35 (0.84–2.21)	0.22
	Other	1.26 (0.31–4.50)	0.73
Type of biliary reconstruction (cf to duct‐to‐duct)	Roux‐en‐Y/HJ	0.44 (0.22–0.82)	0.01

Abbreviations: CIT, cold ischemic time; CVA, cerebrovascular accident; HJ, hepaticojejunostomy; MELD, model for end‐stage liver disease.

**TABLE 3:** Multivariable logistic regression model for anastomotic biliary stricture.

Variable		OR (95% CI)	*p*
Recipient sex: Male		1.44 (0.97–2.17)	0.07
Era of transplant (cf to 2016–2018)			
	2008–2015	3.14 (1.90–5.34)	< 0.01
	2000–2007	3.06 (1.70–5.63)	< 0.01
MELD at transplant		1.03 (1.01–1.06)	0.02
CIT (hours)		1.06 (0.98–1.14)	0.16
Donor age (years)		1.02 (1.01–1.03)	< 0.01
Donor cause of death (cf to trauma)			
	Anoxia	0.61 (0.31–1.19)	0.15
	CVA	0.99 (0.56–1.79)	0.98
	Other	1.35 (0.32–5.11)	0.67
Type of biliary reconstruction (cf to duct‐to‐duct	Roux‐en‐Y HJ	0.47 (0.22–0.93)	0.04

Abbreviations: CIT, cold ischemic time; CVA, cerebrovascular accident; HJ, hepaticojejunostomy; MELD, model for end‐stage liver disease.

**FIGURE 2:**
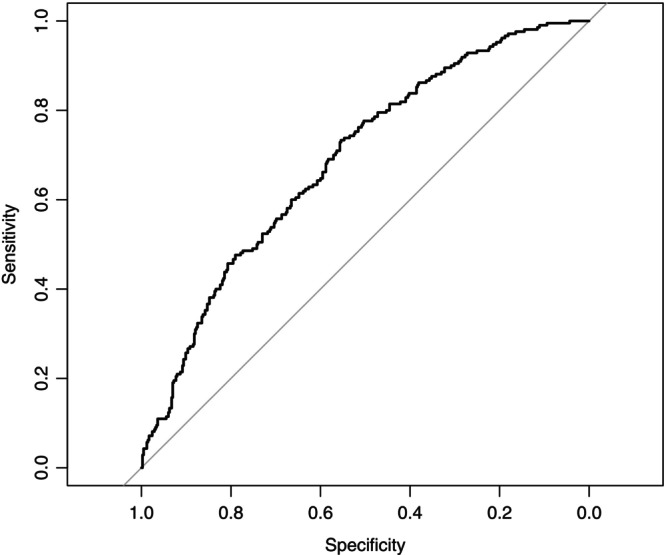
Receiver operating curve: Multivariable model for biliary anastomotic stricture (BAS).

The AUROC for the multivariable model was 0.69 (95% CI: 0.65–0.73), with an internally cross‐validated mean AUROC of 0.66 (SD = 0.04) (Figure 2).

### 3.5 Risk Factors for Early Biliary Anastomotic Stricture

The analysis was repeated with early (1 year) BAS as the outcome measure. There was a total of 519 patients with at least 1 year of follow up, and early BAS was diagnosed in 28% of these patients (*n* = 146). Risk factors for early BAS were similar to the risk factors for BAS overall. Male donor was an additional protective factor, although this was not independently associated with a lower risk of early BAS in the multivariable model (*p* = 0.06) (Tables S1 and S2).

## 4 Discussion

Biliary complications following OLT continue to be a significant source of morbidity and increased healthcare costs [2, 3, 11, 15, 18, 19, 20]. In addition to the technical intricacies of biliary reconstruction, various risk factors are implicated in the development of BAS, including hepatic artery patency, preservation injury, CMV infection, and ABO incompatibility [2, 3, 11, 15, 18, 19, 20].

Some researchers assert that early BAS primarily stems from technical challenges in the anastomosis, especially in the absence of arterial impairment such as hepatic artery thrombosis or arterial stenosis. Late strictures, on the other hand, are linked to fibrotic healing resulting from chronic ischemia or bile leaks [21, 22, 23]. Strategies to reduce BAS incidence involve careful donor and graft handling to avoid injuries, minimizing graft ischemic time and employing precise surgical techniques [24].

Stenting in liver transplantation can be categorized into internal (where the stent is placed and left inside the duct) or external (where the stent is placed in the duct and exteriorized). Internal stents offer the advantage of not creating an opening in the main biliary duct, avoiding potential bile leaks upon removal, and have been proposed by many centers [13, 14, 15, 25]. However, a more recent prospective study has shown that placement of intraductal non‐dissolvable stents does not reduce the incidence of biliary stricture and is associated with difficult endoscopic extraction (19.4%) [26]. The recent development of biodegradable stents may be an area of future research and avoid the difficulties seen with their non‐dissolvable counterparts.

Externalised stents, although having the potential risk of bile leak at the time of removal, have been theorized to reduce the risk of biliary complications. Some authors advocate for routine biliary drainage to reduce stricture rates, with conflicting data generating debate. Randomized trials on T‐tube drainage yield conflicting results, with proponents arguing for its benefits in reducing intra‐biliary pressure (due to anastomotic stenosis or sphincter of Oddi dysfunction), facilitating biliary monitoring for volume and quality, and allowing easy access for biliary imaging in the form of cholangiography. Conversely, opponents highlight increased bile leak rates and hospital readmissions without impacting stricture rates as its pitfalls [5, 7, 8, 9, 10, 17, 26].

Regarding the role of transcystic stenting in liver transplantation, many series have reported its safety and outcomes [13, 14, 27, 28, 29] but there is not a prospective randomized study to confirm these findings. The present study is the largest series reporting the outcomes of transcystic stent use in OLT and among the variables studied, some are in accordance with previous reports [13, 14, 27, 28, 29]. Advanced donor age remains a risk factor that increases BAS odds by almost two times. The mechanism explaining this association remains unclear but may be due to micro‐circulatory changes in the blood supply to bile duct related to aging [30]. On the other hand, younger recipients are protected from BAS development (patients more than 22‐years‐old had a fourfold increase in the chance of having BAS). Better blood supply to the biliary tree and an increased capacity for wound healing could explain this phenomenon.

Established surgical factors implicated in the development of BAS encompass the suturing technique, type of suture, and the method of reconstruction. Through our analysis, we observed that the utilization of continuous suturing elevates the likelihood of BAS by twofold. Additionally, end‐to‐end anastomosis entails a higher risk of BAS compared to all other types of anastomoses. Roux‐en‐Y hepaticojejunostomy reconstruction demonstrated a trend towards reduced risk of BAS when contrasted with end‐to‐end anastomosis.

Our data deviates from specific studies that indicate a lower occurrence of stenosis in patients with end‐to‐end choledocho‐choledochostomy [28]. It is essential to recognize that variations in the definition of biliary stricture may have contributed to the observed discrepancies among the various studies. Interestingly, the presence of a transcystic externalised trans‐anastomotic stent did not show a correlation with the development of BAS across all anastomosis types.

It is imperative to acknowledge the limitations of our study, given its singular center focus, long period of study observation, and the absence of external validation for our findings. Nevertheless, the study's strength lies in the consistency of indications, standardized surgical techniques, and the uniform application of a transcystic externalized trans‐anastomotic biliary stent over the study period.

## 5 Conclusions

Despite the inherent limitations of this single‐center, retrospective study, our findings suggest that the routine use of transcystic externalised trans‐anastomotic biliary stenting during liver transplantation does not change the incidence of biliary anastomotic stricture and its ongoing use cannot be recommended.

## Conflicts of Interest

The authors declare no conflicts of interest.

## Supporting information


**TABLE S1.** Univariate analysis of risk factors for early (< 1 year)anastomotic bile duct stricture.
**TABLE S2:** Multivariable model for early (< 1 year) anastomotic bile duct stricture.

## Data Availability

The data that support the findings of this study are available on request from the corresponding author. The data are not publicly available due to privacy or ethical restrictions.
