# Supplementary material for: To Stent or Not to Stent: Is It a Question? Routine Trans‐Cystic Stenting Does Not Reduce Biliary Anastomotic Strictures Post‐Liver Transplantation
Source: ANZ J Surg. 2025 Jun 23;95(9):1932–9. doi: 10.1111/ans.70224 (PMC12484385; doi:10.1111/ans.70224)
Supplement: Supplementary file 1 — TABLE S1. Univariate analysis of risk factors for early (< 1 year)anastomotic bile duct stricture. TABLE S2: Multivariable model for early (< 1 year) anastomotic bile duct stricture. [file ANS-95-1932-s001.docx]

***Supplementary table 1:*** *Univariate analysis of risk factors for early (<1 year)anastomotic bile duct stricture*

| **Variable** |  | **OR (95%CI)** | **p** |
| --- | --- | --- | --- |
| Recipient sex: Male |  | 1.43 (0.94-2.22) | **0.09** |
| Era of transplant (cf to 2016-2018) |  |  | **0.05** |
|  | 2008-2015 | 2.00 (1.12-3.75) | **0.02** |
|  | 2000-2007 | 1.99 (1.01-3.92) | **0.03** |
| MELD at transplant |  | 1.04 (1.01-1.07) | **0.02** |
| Donor age (years) |  | 1.02 (1.01-1.03) | **<0.01** |
| Donor sex: Male |  | 0.72 (0.49-1.05) | **0.09** |
| Donor cause of death (cf to trauma) |  |  | **<0.01** |
|  | Anoxia | 0.56 (0.26-1.19) | 0.13 |
|  | CVA | 1.46 (0.84-2.64) | 0.19 |
|  | Other | 0.49 (0.02-3.13) | 0.52 |
| Type of biliary reconstruction (cf to duct-to-duct | Roux-en-Y HJ | 0.35 (0.13-0.78) | **<0.01** |

CIT, cold ischaemic time; CVA, cerebrovascular accident HJ, hepaticojejunostomy; MELD, model for end-stage liver disease

***Supplementary table 2****: Multivariable model for early (<1 year) anastomotic bile duct stricture*

| **Variable** |  | **OR (95%CI)** | **p** |
| --- | --- | --- | --- |
| Recipient sex: Male |  | 1.70 (1.04-2.82) | **0.04** |
| Era of transplant (cf to 2016-2018) |  |  |  |
|  | 2008-2015 | 2.20 (1.15-4.47) | **0.02** |
|  | 2000-2007 | 2.33 (1.13-5.02) | **0.03** |
| MELD at transplant |  | 1.02 (0.98-1.05) | 0.28 |
| Donor age (years) |  | 1.01 (1.00-1.03) | 0.10 |
| Donor sex: Male |  | 0.67 (0.43-1.04) | 0.07 |
| Donor cause of death (cf to trauma) |  |  |  |
|  | Anoxia | 0.61 (0.31-1.19) | 0.10 |
|  | CVA | 0.99 (0.56-1.79) | 0.89 |
|  | Other | 1.35 (0.32-5.11) | 0.48 |
| Type of biliary reconstruction (cf to duct-to-duct | Roux-en-Y HJ | 0.47 (0.22-0.93) | **0.03** |

CIT, cold ischaemic time; CVA, cerebrovascular accident HJ, hepaticojejunostomy; MELD, model for end-stage liver disease
